# Antibiotics Usage in Special Clinical Situations

**DOI:** 10.3390/antibiotics13010034

**Published:** 2023-12-29

**Authors:** Stefano Di Bella

**Affiliations:** Clinical Department of Medical, Surgical, and Health Sciences, Trieste University, 34127 Trieste, Italy; stefano932@gmail.com

Medicine and the treatment of infectious diseases are increasingly focused on patient-tailored diagnostics and therapy. There are various aspects to consider for an optimized therapy, both in terms of efficacy and toxicity, as well as in the context of antimicrobial stewardship [1]. In the last decade, there has been ample evidence supporting the consideration of “short” regimens for common infectious pathologies (e.g., bloodstream infections, pneumonia, spondylodiscitis) [2,3,4]. Simultaneously, especially for challenging bacteria (e.g., *Pseudomonas*, *Enterococcus*) [5,6], studies on combination therapy (typically involving fosfomycin-containing regimens) have revealed exciting potential. The role of therapeutic drug monitoring is gaining prominence [7], not only in determining if pharmacological levels are adequate but also in revising drug dosages, potentially minimizing adverse effects. Discussing special clinical situations in infectious diseases allows us to review current clinical practices in light of advancing knowledge.

This Special Issue, for which I served as the Guest Editor, highlighted several crucial points, which I will briefly outline below:

Osteomyelitis:

(1) Evidence suggests that short-term therapy is non-inferior to long-term therapy, particularly in vertebral osteomyelitis [8].
(2) Increasing evidence indicates that sequential therapies (intravenous → oral) can be employed for bone infections, reducing hospitalization duration, improving patient convenience, and lowering healthcare costs without compromising clinical success [9]. The limitation lies in the ability to choose oral options when there is sensitivity to molecules with high bioavailability.
(3) For many years, the use of rifampicin in *Staphylococcus*-related osteomyelitis has been widely considered as part of a combination regimen [10,11]. This is attributed to its well-known pharmacokinetic/pharmacodynamic properties, antibiofilm activity, and reported clinical evidence. While this practice is common among infectious disease specialists, revisiting the evidence reveals a need for more robust studies on larger cohorts in the future.

Vancomycin-resistant *Enterococcus faecium* infections:

(1) There is no standardized therapy for vancomycin-resistant *Enterococcus faecium* (VRE) infections. Typically, reliance is placed on linezolid or daptomycin in combination with a beta-lactam. However, these regimens have significant limitations, illustrated by two examples: linezolid is not the optimal drug for bloodstream infections, and resistance to daptomycin among VRE is increasing [11].
(2) In this context, two drugs (one old and one new) are interesting to consider for VRE infections: fosfomycin and oritavancin. The rationale behind this combination is not only to synergize with each other but also to cover almost all potential clinical infection sites.
(3) Lagatolla et al. demonstrated in vitro and in vivo (using *Galleria mellonella*) that the combination of oritavancin and fosfomycin increased drug susceptibility, showing a synergistic effect in 80% of isolates and an additive effect in the remaining isolates. Interestingly, the combination restored fosfomycin susceptibility in 85% of fosfomycin-resistant isolates. In the animal part of the study, the authors demonstrated a higher survival rate of larvae treated with combination therapy compared to monotherapy (fosfomycin or oritavancin alone).

Diverticular disease:

(1) In patients with diverticular disease, it would be advantageous to use drugs that can target the specific causative bacteria without, as much as possible, affecting the rest of the intestinal flora.
(2) While acknowledging that the perfect drug does not exist, rifaximin possesses unique characteristics that make it an interesting molecule for patients with diverticular disease. These characteristics include low systemic absorption, high stool concentration, and specific antibacterial properties.
(3) Piccin et al. reviewed the literature evidence in patients with diverticulosis, symptomatic uncomplicated diverticular disease, acute diverticulitis (primary and secondary prophylaxis), and uncomplicated acute diverticulitis.

Sulbactam/durlobactam for *Acinetobacter* infections (focus on resistances):

(1) *Acinetobacter* is a bacterium for which available antimicrobial options are minimal [12].
(2) The latest pharmacological discovery, cefiderocol, did not show brilliant results in randomized clinical trials for *Acinetobacter* infections [13]. A new finding has emerged: sulbactam/durlobactam. This drug completed a phase 3 trial that proved non-inferior to colistin for 28-day all-cause mortality in *Acinetobacter* hospital-acquired bacterial pneumonia, ventilator-associated bacterial pneumonia, or bloodstream infections [14].
(3) Principe et al. conducted a systematic review of in vitro studies reporting *A. baumannii* resistances against sulbactam/durlobactam, revealing various resistance patterns.

The impact of macrolides on children’s gut flora:

(1) The protective role of a “healthy” microbiota is increasingly being recognized. Understanding and comparing the gut-disrupting effects of different antibiotic molecules is crucial. In pediatric clinical practice, macrolide antibiotics at low doses are often used off-label for their prokinetic action [15].
(2) Thavamani et al. conducted a case–control study on pediatric patients with feeding intolerance, analyzing the bacterial and fungal microbiome of cases (patients receiving erythromycin) and controls (patients not receiving erythromycin).
(3) The study found significant differences in beta diversity between the groups for the mycobiome at the species level, with an increase in the relative abundance and prevalence of many fungi in the erythromycin-exposed group.

Antibiotics for diabetic foot infections:

(1) Diabetic foot infections present complex therapeutic challenges, requiring simultaneous targeting of Gram-positive, Gram-negative, and anaerobic bacteria [16,17].
(2) Wright et al. provided an overview of systematic reviews, including 29 individual studies published between 2015 and 2022.
(3) The heterogeneity observed in the studies was high, and no significant clinical differences were found when comparing fluoroquinolones with piperacillin/tazobactam or amoxicillin/clavulanate. Similarly, no differences were found when comparing ertapenem to piperacillin/tazobactam. However, compared to ertapenem, tigecycline did not demonstrate non-inferiority and was associated with a higher likelihood of adverse events than ertapenem (with or without vancomycin).

## Data Availability

No research data were generated for this editorial.
